# High-Throughput Liquid Chromatography–Tandem Mass Spectrometry Quantification of Glycosaminoglycans as Biomarkers of Mucopolysaccharidosis II

**DOI:** 10.3390/ijms21155449

**Published:** 2020-07-30

**Authors:** Junhua Wang, Akhil Bhalla, Julie C. Ullman, Meng Fang, Ritesh Ravi, Annie Arguello, Elliot Thomsen, Buyankhishig Tsogtbaatar, Jing L. Guo, Lukas L. Skuja, Jason C. Dugas, Sonnet S. Davis, Suresh B. Poda, Kannan Gunasekaran, Simona Costanzo, Zachary K. Sweeney, Anastasia G. Henry, Jeffrey M. Harris, Kirk R. Henne, Giuseppe Astarita

**Affiliations:** Denali Therapeutics, Inc., 161 Oyster Point Blvd, South San Francisco, CA 94080, USA; bhalla@dnli.com (A.B.); ullman@dnli.com (J.C.U.); fang@dnli.com (M.F.); ravi@dnli.com (R.R.); arguello@dnli.com (A.A.); thomsen@dnli.com (E.T.); tsogtbaatar@dnli.com (B.T.); guo@dnli.com (J.L.G.); skuja@dnli.com (L.L.S.); dugas@dnli.com (J.C.D.); davis@dnli.com (S.S.D.); suresh_poda@eisai.com (S.B.P.); kannan@dnli.com (K.G.); costanzo@dnli.com (S.C.); sweeney@dnli.com (Z.K.S.); henry@dnli.com(A.G.H.); jharris@dnli.com(J.M.H.); henne@dnli.com (K.R.H.); gastarita@gmail.com (G.A.)

**Keywords:** mucopolysaccharidosis, iduronate 2-sulfatase, liquid chromatography-tandem mass spectrometry, glycosaminoglycans, heparan sulfate, dermatan sulfate, cerebrospinal fluid

## Abstract

We recently developed a blood–brain barrier (BBB)-penetrating enzyme transport vehicle (ETV) fused to the lysosomal enzyme iduronate 2-sulfatase (ETV:IDS) and demonstrated its ability to reduce glycosaminoglycan (GAG) accumulation in the brains of a mouse model of mucopolysaccharidosis (MPS) II. To accurately quantify GAGs, we developed a plate-based high-throughput enzymatic digestion assay coupled with liquid chromatography–tandem mass spectrometry (LC-MS/MS) to simultaneously measure heparan sulfate and dermatan sulfate derived disaccharides in tissue, cerebrospinal fluid (CSF) and individual cell populations isolated from mouse brain. The method offers ultra-high sensitivity enabling quantitation of specific GAG species in as low as 100,000 isolated neurons and a low volume of CSF. With an LOD at 3 ng/mL and LLOQs at 5–10 ng/mL, this method is at least five times more sensitive than previously reported approaches. Our analysis demonstrated that the accumulation of CSF and brain GAGs are in good correlation, supporting the potential use of CSF GAGs as a surrogate biomarker for brain GAGs. The bioanalytical method was qualified through the generation of standard curves in matrix for preclinical studies of CSF, demonstrating the feasibility of this assay for evaluating therapeutic effects of ETV:IDS in future studies and applications in a wide variety of MPS disorders.

## 1. Introduction

Mucopolysaccharidosis type II (MPS II), or Hunter Syndrome, is an X-linked lysosomal storage disease caused by deficiency of iduronate 2-sulfatase (IDS), a lysosomal enzyme involved in the catabolism of the glycosaminoglycans (GAGs) heparan sulfate (HS) and dermatan sulfate (DS) [1]. Approximately two-thirds of MPS II patients display the neuronopathic phenotype, characterized by progressive cognitive impairment, behavioral symptoms, and a decreased life span, with death typically occurring within the second decade of life [1]. 

The progressive accumulation of undegraded GAGs (including HS/DS, and keratan sulfate (KS) which has been reported recently [2,3]) in lysosomes is a molecular hallmark of MPS diseases, which ultimately results in cell, tissue, organ dysfunction, and bone deformities [4]. GAGs are a major class of extracellular matrix biomolecules [5] that are secreted into the circulatory system (blood), urine and CSF, and thus have been widely evaluated as a biomarker of primary storage in preclinical models of MPS as well as in MPS patients. There is a growing body of evidence to support the use of urinary GAGs as predictive biomarkers of MPS, as well as treatment responsive biomarkers in MPS II patients on enzyme replacement therapy (ERT) [3,6,7,8]. The field has previously acknowledged that there is a lack of relationship between GAG levels across different organs or biofluids [9]. The correlation between biofluid and its relevant tissue (e.g., urinary vs. kidney GAGs), however, is much more significant, providing a unique perspective of using the biochemical changes of GAGs in specific biofluid and corresponding tissue as a biomarker for the characterization of MPS subtypes and treatment. To accurately evaluate the efficacy of potential blood–brain barrier (BBB)-penetrating ERTs, including our enzyme transport vehicle fused to iduronate 2-sulfatase (ETV:IDS) in preclinical studies [10,11] using an MPS II mouse model, a robust and highly sensitive method is required to quantify GAGs in brain and biofluids.

CSF is in constant exchange with the interstitial fluid of the brain, making it a unique medium to investigate the biochemical changes in the central nervous system (CNS) [12]. Measuring CSF GAGs in preclinical mouse models is relatively more challenging than other biofluids or tissues due to the limitation of sample volume in combination with the low concentration of GAGs. Analytical technology has advanced significantly and become increasingly amenable to reliably measuring GAGs in CSF. Indeed, the field has progressed significantly from the first measurement of acidic GAG hexuronic acid, which required several milliliters of Hunter-Hurler’s syndrome CSF [12], to a recent study [13] that used only a few microliters of CSF to evaluate heparan sulfate (HS) content as a potential biomarker for evaluating brain GAG accumulation in a MPS II mouse model.

This advancement has been directly enabled by the technological advances in liquid chromatography mass spectrometry (LC-MS/MS). Over the past decade, LC-MS/MS has become the preferred platform to analyze chemically or enzymatically degraded oligosaccharides from broad classes of GAGs [3,8,13,14,15,16,17,18,19,20,21,22,23,24,25,26,27]. Acid hydrolysis of GAGs coupled with LC-MS/MS quantification is a highly sensitive method that has been successfully applied to biological samples from mouse models of MPS and MPS patient samples [19]. The acid hydrolysis chemical reaction, via methanol [8,13,28,29], ethanol or butanol [22], depolymerizes the complex GAGs into discrete units detected via mass spectrometry. This method has the ability to simplify the complex and heterogeneous GAGs into reduced disaccharide species which can be helpful for analysis but potentially oversimplifies the chemical biology. For example, through desulfation/deacetylation and dialkylation processes all variants of HS will be reduced to a single species with a uniform disaccharide backbone. This process concentrates the molar amounts of disaccharide analytes tens to hundreds of times higher than the molar number of intact GAGs, which directly improves the detection limit because of the molar enrichment. Enzymatic depolymerization can achieve similar sensitivity enhancement, but more importantly also retains the native chemical diversity [26,30]. Enzymatic digestion of the GAGs releases unmodified disaccharides that are compositionally informative and unique to the enzymes linked to different types of MPS diseases [31]. In this process, heparan sulfate (HS) and dermatan sulfate (DS) polysaccharides are digested with heparinases and chondroitinase-B, respectively, to release HS and DS disaccharides. This process generates products that maintain both their original chemical modifications and structural diversities inherent to the endogenous molecules.

In this paper, we describe a high-throughput assay to measure HS and DS GAG disaccharides. The method combines plate-based enzymatic digestion with hydrophilic interaction chromatography (HILIC)-tandem mass spectrometry (HILIC-MS/MS). The method was applied to simultaneously quantify HS and DS derived disaccharides in peripheral tissues, brain, CSF, and isolated brain cells (neurons, astrocytes and microglia) from the brains of MPS II model mice. The method offers high sensitivity to enable the detection of GAGs in limited samples, e.g., FACS-sorted neurons from brain, and minimal CSF volume from MPS II mice. This bioanalytical method was qualified using surrogate CSF matrix, and it achieved a LOD of 3 ng/mL and a LLOQ of 10 ng/mL using only 3 µL of CSF, based on calibration curve analysis in CSF for preclinical studies. These results demonstrate the utility of this assay to evaluate brain and CSF GAGs to assess ETV:IDS therapeutic efficacy, and establishes CSF GAG levels as a surrogate for brain GAG levels [11] in the MPS II mouse model.

## 2. Results and Discussion

### 2.1. Assay Development 

#### 2.1.1. Chemical Hydrolysis vs. Enzymatic Digestion 

Chemical hydrolysis and enzyme-based digestion have their respective advantages in GAGs analysis. The chemical hydrolysis method has different variants, for example, nitrous acid [26], HCl methanolysis [29] and HCl butanolysis [22]. The products of chemical hydrolysis are rather simple, as shown in Figure S1. However, one of the disadvantages is that the original epimeric nature of the uronic acid and the information related to the N-acetylation or N-sulfation are lost during the desulfation and deacetylation by acid hydrolysis. In contrast, enzymatic digestion cleaves the hexosamine-hexuronic acid glycosidic bonds, preserving the stereochemistry of anomeric carbon [31] and the N-acetylation or N-sulfation modifications. Thus, enzymatic digestion offers the possibility to investigate the biological function of GAGs that are pertinent to their structure. To understand which approach would be most robust for our purposes, we tested both strategies side by side.

First, we evaluated chemical hydrolysis using both methanolysis and butanolysis for generating disaccharides based on previously published methods [22]. However, to our surprise, butanolysis yielded two HS-derived peaks with the same mass instead of a single peak as was expected. DS hydrolysis, via acid methanolysis, yielded at least five distinct peaks with the same mass in both DS standards as well as in biological samples such as CSF (Figure S2), which was observed by Menkovic et al. [8] in MPS patients’ urine samples. Additionally, we performed an MS/MS experiment that confirmed that those isomeric peaks were identical molecules (although, MS/MS spectra do not provide the stereochemical structural confirmation). Hydrolysis using heavily labeled methanol and butanol confirmed the same product structure (Figures S3 and S4). Taken together, the acid hydrolysis method presented technical ambiguity that introduces a significant challenge for understanding the correct peak identity and ultimately determine which peak(s) accurately reflect the HS and DS in biological samples. Based on these results, no further consideration was given to the chemical hydrolysis method in our studies.

To effectively quantify this endogenous structural heterogeneity, we pursued the enzymatic digestion of GAGs. The enzymatic digestion depolymerizes high molecular weight GAGs to M.W. 350-600 Da HS and DS disaccharides with unsaturated bonds on Δ-4,5-unsaturated hexuronic acid for LC-MS/MS analysis [26,30], which allows enrichment of the molecular amounts for improved sensitivity. To shorten the sample processing time for large preclinical studies, derivatization or labeling strategies were intentionally avoided in order to maintain the highest reproducibility. Heparinases I, II, III and Chondroitinase B were used as a mixture to release HS and DS disaccharides simultaneously from GAG polysaccharides in a single sample preparation.

#### 2.1.2. Analytical Method Workflow

**Figure 1:**
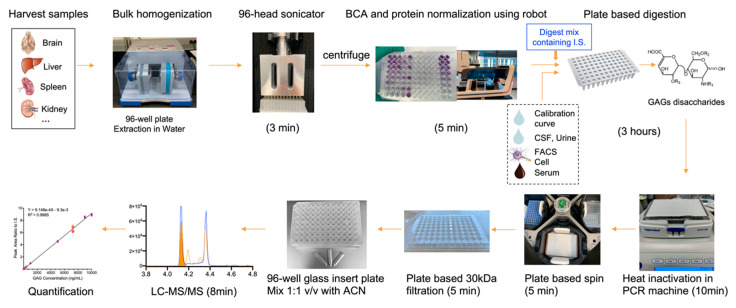
LC-MS/MS GAGs analysis using a robotic workstation-assisted and plate-based sample preparation workflow.

Figure 1 shows the workflow schematic implemented for LC-MS/MS GAGs analysis (unless otherwise specified, the terms GAGs, HS (e.g., D0A0, D0S0) and DS (D0a4) refer to their respective disaccharides throughout this paper). Semi high-throughput sample preparation was achieved through the use of a robotic workstation and a plate-based workflow. This process was adapted for various sample types such as tissues and biofluids, as well as standard curves and quality controls (QCs). Tissue, biofluids and standard curve solutions were harvested into 96-well microplates to enable downstream plate-based processing. Samples were introduced at different steps of the workflow depending on the sample type. For example, tissue samples (brain, liver, kidney, heart, spleen, and lung) were homogenized in water using a Tissuelyser^®^ followed by sonication in a 96-head tip sonicator. Biofluid samples (e.g., CSF, serum and urine or FACS sorted cells) were not subjected to the homogenization and protein normalization steps. The final internal standard (I.S.) concentration was spiked at 20 ng/sample for all samples, including the calibration solutions. The use of robotic liquid handling significantly reduced I.S. variability by increasing I.S. methanol solution transferring speed, which effectively minimized evaporation. A heat activated plate sealer was also employed to seal the plate in order to minimize further evaporation and loss of volume during the digestion reaction. The enzymatic reaction was allowed to proceed for 3 h at 30 °C to complete digestion, and heat inactivated for 10 min. Following heat inactivation, the digested material was cleared by a 5 min spin before passing the supernatant through a 30 k Da MWCO filter microplate for final cleanup. The flow-through was transferred to a LC-MS compatible 96-well plate with glass inserts, and an equal volume of acetonitrile was added by robotic liquid handling, followed by LC-MS/MS. All the steps outlined above were enabled by the use of a robotic multi-channel head to transfer samples and reagents. The entire process was streamlined using a plate-based format from the beginning to the end, which considerably expedited the overall sample processing time as well as reduced errors that could be encountered by operating in an individual tube or vial format.

#### 2.1.3. Contribution of Ca^2+^ and EDTA to LC-Based Separation

The enzymatic digestion procedure requires calcium (Ca^2+^) in the reaction buffer to catalyze the activity of the digestion enzymes. Thereafter, excess EDTA was added in the final step to chelate Ca^2+^. Addition of EDTA in the sample preparation protocol was found to be critical for the BEH amide LC column (described below) to maintain its separation efficiency as well as to maintain longevity; otherwise, the column performance degraded relatively quickly. Based on our observations, we speculate that the presence of free Ca^2+^ is potentially harmful to the amide column. The EDTA and Ca^2+^ concentration carried over from the digestion step worked well, and no further optimization was performed.

#### 2.1.4. The MWCO Filterplate for Sample Cleanup

After digestion, heat inactivation and initial spin, the sample containing disaccharide products was filtered through a 96-well, 30 kDa MWCO filter microplate made of regenerated cellulose. This material was critical for maintaining a clean LC profile downstream. To test this, a flow-through of water/acetonitrile mixture using this MWCO filter microplate was injected onto a high-resolution mass spectrometer, and it showed no leakage of any PEG-like polymers from m/z 300 to 4000. In contrast, for the same mixture using other polyethersulfone or polypropylene-based filter microplates, overwhelmingly high PEG polymer peak clusters from m/z 400 to 1800 were detected. This showed that sample flow-through from regenerated cellulose membrane filters is cleaner than from other filter materials. In addition, the recovery of HS/DS internal standard was nearly ~100% both with and without sample matrix. Thus, this MWCO filter microplate is an effective and efficient tool for sample cleanup.

#### 2.1.5. A New and Fast HILIC Method for Simultaneous HS and DS Analysis 

Resolving and measuring the isomeric HS and DS disaccharides simultaneously with high precision is challenging, but also necessary for accurate GAG quantification. Enzymatically digested GAG disaccharides are polar molecules, and several HILIC [26,32] or graphitic carbon columns [24,30] have been established for the analysis of HS [26] or HS and DS simultaneously [30,32]. However, these methods are either very lengthy, with 40 to 60 min run time per sample [24,26,32], or the separation performance has not been clearly demonstrated [30]. We prefer this HILIC method over some existing ion pairing (IP) LC approaches [14] since the HILIC mobile phases are mass spectrometer (MS) compatible, while IP agents, tributylamine for example, are widely known to be sticky to the MS and to cause significant ion signal suppression.

**Figure 2:**
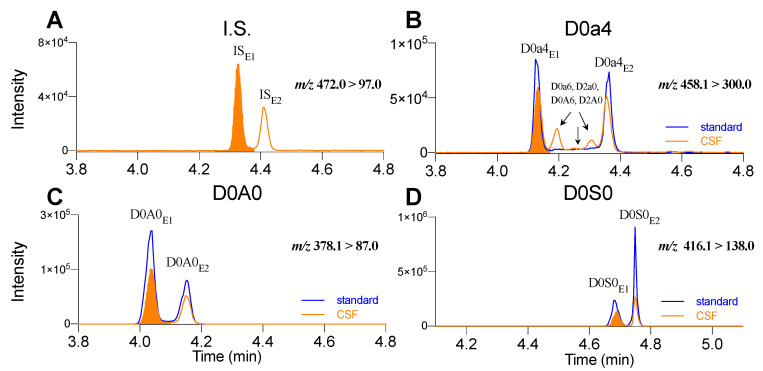
Representative chromatograms of internal standard and disaccharide peaks from enzymatically digested CSF sample of an *Ids* KO mouse in a single run. (**A**) internal standard (I.S.) ∆4UA-2S-GlcOEt-6S, (**B**) D0a4, (**C**) D0A0, and (**D**) D0S0. Each species shows two epimers designated as E1 and E2. Shaded orange peaks are the E1s that were used to represent the species for quantification. Blue traces are the authentic standards in a separate run for D0a4, D0A0 and D0S0, respectively. LC-MS conditions refer to experiment section.

One successful example of rapid separation of hydrolyzed HS and DS was established using a short (5 cm) Waters ACQUITY BEH amide column by Menkovic et al. [8]. Here, we developed multiple HILIC gradients by using a 15-cm BEH amide column for the analysis of enzymatically digested disaccharides. An 8-min isocratic gradient was able to achieve baseline separation of the analytes without the need for very long equilibration process for a new column. The method achieved baseline separation and accurate profiling of HS and DS (Figure 2), at two levels: resolution of the epimeric pairs for each HS or DS, and resolution of the isomeric pairs between HS and DS in complex matrices. An example of LC peak overlay for *Ids* KO; TfR^mu/hu^KI (herein referred to as *Ids* KO) mouse CSF sample of the major HS and DS species is shown in Figure 2. 

This column was also suitable to run a higher throughput (4.5 min as compared to 8 min) gradient for GAG separation. However, in order to achieve the same level of separation with a shorter run time, the column required equilibration in the form of multiple pre-run injections (approximately 50 blank injections). To overcome this, we injected a pooled matrix sample and were thus able to expedite the process by reducing the injection numbers by ~30%. This is presumably because the processed biological sample has all the properties needed to age the column, i.e., GAGs, matrix, and the previously discussed EDTA. The LC gradient of this method and an example separation from a FACS-isolated neuron is shown in Figure S5.

#### 2.1.6. GAG Compositions in Preclinical Samples

GAG polysaccharides are typically made up of 20 to 200 sugar units in length (M.W.~10k–100kDa), making them relatively challenging to quantify at the intact level. HS and DS (the most relevant GAG species in MPS II) are comprised of variable lengths and exhibit considerably high structural heterogeneity (48 potential disaccharide units, in theory) due to the various degrees of epimerization, sulfation, and acetylation. Lawrence et al. [33] introduced a new nomenclature called disaccharide structural code (DSC) for designating the disaccharide subunit structure of all GAGs as a way to describe their compositions and linear sequences. Enzymatic degradation can keep all the composition and modification information for the disaccharides, which is one of the reasons that this method was appealing to us. The major HS and DS/CS disaccharide species codes, structures, molecular masses as well as multiple reaction monitoring (MRM) transitions are shown in Figure S6. In addition to the species diversities, the level of different GAG species also varies in different organisms, for example in mouse kidney, the dominating GAG is HS representing 74–75% of total GAGs, the CS/DS content is 18–20% and hyaluronic acid (HA) represents approximately 6% of the total [34]. Some studies have reported that the disaccharides D0A0 and D0S0 are the most abundant HS species in mouse and human, varying from 70% to 85%, and D0a4 is about 95% among the DS family [26,30,35]. Indeed, D0A0 and D0a4 have been used as proxy analytes in serum GAG measurements to represent total GAGs [36], and the study showed that D0A0 and D0a4 levels correlate well with the total HS and DS disaccharide levels, respectively [36]. In our studies, the combined levels of D0A0, D0S0, and D0a4 was used to represent the sum of GAGs for biomarker analysis. 

#### 2.1.7. GAG Epimer and Isomer Separation and Individual Species Quantification

Each HS or DS disaccharide consists of two natural epimeric species due to the anomeric carbon in the uronic acid sugar. A previous study using a Thermo^®^ amide HILIC column suggested that higher column temperature (~60 °C) could merge the two epimeric peaks into one [26]. However, we were unable to replicate this in our studies, probably because of the difference in column chemistry, even though both columns are amide-based. Using our LC method, all disaccharides were detected as two epimeric peaks, the same as literature reported previously [24]. In preclinical mouse as well as human samples, we identified three major disaccharides, D0A0 (HS), D0S0 (HS), and D0a4 (DS) as the most abundant GAG disaccharides in the enzymatically digested samples. We successfully isolated the first epimeric peak for each individual species. Their identities were all confirmed by synthetic standards (Figure 2) and by high-resolution MS/MS spectra. Upon confirming their unique identities, disaccharide species were thereafter quantified by the ratio of the peak area to the I.S. peak area. Area ratios were further normalized to sample input amount, either by total protein for tissue, sample volume for biofluids, or cell number. The I.S. used in these studies is a synthetic disaccharide, ∆4UA-2S-GlcOEt-6S, which has been widely used in enzymatic LC-MS analyses [14,30] and elutes as two epimeric peaks IS_E1_ (4.3 min) and IS _E2_ (4.4 min) as shown in Figure 2A. 

Once the epimeric and isomeric separation was confirmed with the disaccharide standards, the next step was to identify which disaccharide species we could reliably quantify in our samples. ∆UA-GalNAc4S (D0a4) represents the most abundant DS disaccharide species according to the literature [26,30,35] and is among several DS and HS stereoisomers, including D0a6, D2a0, D0A6, D2A0. The presence of isomers and epimers makes it particularly challenging to separate out a pure D0a4 peak for quantitation purposes. By checking structures (e.g., sulfation position) and previous reports, we found D0a4 was distinguishable from its isomers by the transition m/z 458.1 > 300.0, which is more specific to the sulphated S4 position [24,30], whereas the S0 and S6 are detected by m/z 458.1 > 97.0 or 458.1 > 282.0. In addition, fine-tuning the LC gradient ensured complete resolution of the first epimeric peak of D0a4 from the rest of the isomeric peaks that largely coeluted with it (Figure 2B). We found that in biological samples, particularly in CSF, D0a4 was remarkably higher than the total level of the other isomers. As such, the total amount of D0a4 was quantified by normalizing the first epimeric peak of D0a4 to the first epimeric peak of I.S. (i.e., D0a4_E1_/IS_E1_) to reduce potential contamination from other isomers and epimers. We applied a similar approach to differentiate the Glu/Gal isomers, ∆DiHS ∆UA-GluNAc (D0A0) and ∆DiDS ∆UA-GalNAc (D0a0) which share the same molecular formula. However, we found the transition m/z 378.1 > 87.0 unique to D0A0, as it produced much higher signal than using m/z 378.1 > 175.0, which has been more frequently used elsewhere for D0a0 [30]. To be consistent with D0a4, the total amount of D0A0 was represented by normalizing the first epimeric peak of D0A0 to the first epimeric peak of I.S. (i.e., D0A0_E1_/IS_E1_) (Figure 2C). Similarly, for another highly abundant disaccharide HS species, ∆UA-GluNS (D0S0), we found no interfering peak in biological samples (Figure 2D) and it was included with D0a4 and D0A0 to represent the sum of GAGs in this study. The acquisition parameters on QTRAP 6500+ MS and retention time (RT) for I.S. and all three GAG species are summarized in Table S1.

### 2.2. Assay Qualification: Linear Range, Sensitivity, Accuracy and Precision

The assay was qualified with a ‘fit-for-purpose’ approach for quantifying GAGs in CSF samples from preclinical studies. Ultrapure authentic standards D0A0, D0S0 and D0a4 and I.S. (4UA-2S-GlcNCoEt-6S) were purchased from Iduron. To prepare calibration curve and quality control (QC) samples, the standard stock solutions were spiked in pooled human CSF to desired concentration levels (see Section 3.6) and were prepared in the same manner as biological samples, with the exception that no enzymes were added to the digest mix. This was done to prevent the digestion of endogenous GAGs in matrix.

**Figure 3:**
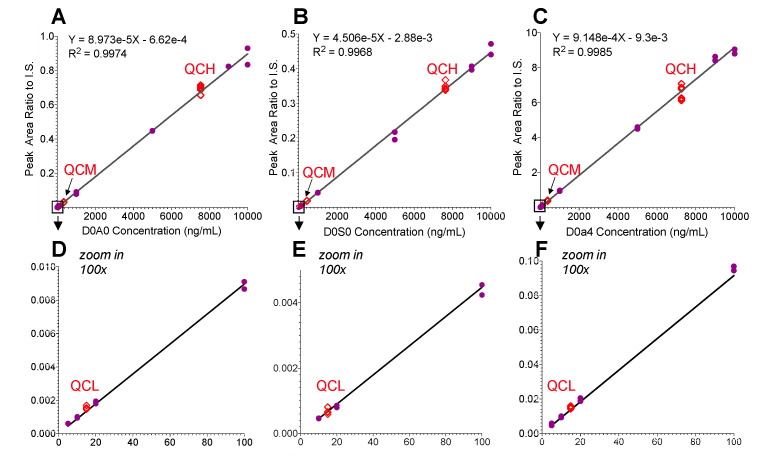
Calibration curves for D0A0, D0S0 and D0a4 based on the ratio of peak area to I.S. area using undigested human CSF as matrix. (**A**–**C**) Dynamic range 5–10,000 ng/mL, (**D**–**F**) inset for dynamic range 5–100 ng/mL. I.S. concentration 20 ng/sample. GAG concentration levels: 5, 10, 20, 100, 1000, 5000, 9000 to 10,000 ng/mL. QCH = 7500 ng/mL, QCM = 300 ng/mL, QCL = 15 ng/mL. Total QCs *n* = 6. Three (3) µL of standard solutions were used to mimic the mouse CSF sample preparation procedure.

Figure 3 shows the calibration curves for the three disaccharides by using their area ratios to I.S. (y-axis) against concentration (x-axis). The regression correlation coefficients (R^2^) for D0A0, D0S0 and D0a4 were 0.997–0.998. Based on less than 20% (CV) acceptance criteria for LLOQ, the method quantitation dynamic range for the standard disaccharides were determined to be from 5 ng/mL to 10,000 ng/mL for D0a4 and D0A0, and 10 ng/mL to 10,000 ng/mL for D0S0 (Figure 3). The LOD, however, was ~3 ng/mL for D0a4 and ~5 ng/mL for D0A0 and D0S0. The assay qualification results, including inter-assay and intra-assay precision (% CV) and accuracy (% bias) for standards and QCs are summarized in Table 1. For determining digestion consistency, we digested a matrix QC sample (pooled CSF) in three independent experiments and calculated the % CV to be between 7.5–10.5%.

There are significant challenges in measuring GAGs from biofluids such as CSF, due to the low GAG concentrations in CSF and the sample volume limitation in mice, as well as from a pediatric population such as MPS II patients. The technology advances on both mass spectrometer and LC sides, have made it possible to measure GAGs in smaller CSF volumes while maintaining high robustness and accuracy. The reported concentration of GAGs and their differences in normal and disease states are quite consistent over the past decades, which were mostly in the range of around 200 ng/mL and 1,000-10,000 ng/mL range, respectively, as shown in Table 2. We find that the LOD and LLOQ in our method are superior than other reported methods in terms of the mass limit of detection (LOD) and the LLOQ. In comparison to published data for CSF (Table 2), our method has similar or better LLOQ, but uses much smaller (5- to 30-fold) CSF volumes [14], and our LOD is at least 7-fold better than that of other studies that use similar volumes of mouse CSF, for example, ref. [13]. The high sensitivity enabled us to detect individual GAG species in limited samples, e.g., neurons and microglia isolated from brain, and minimal CSF volume from mice.

### 2.3. Quantification of GAGs in a Mouse Model of MPS II and MPS II Patient Samples

#### 2.3.1. GAGs in FACS-Isolated Brain Cell Types 

Fluorescence-activated cell sorting (FACS) was used to isolate selected cell populations from whole brain tissues, including neurons, astrocytes and microglia, to better understand the cell-type specific distribution of GAGs [11]. Depending on the brain region, sorting duration and conditions, we typically obtained in the range of 100,000 to 250,000 neurons and 200,000 to 350,000 microglia and astrocytes, which provided sufficient signal to quantify both HS and DS. Cell pellets post sorting were subjected to enzymatic digestion as described above before quantification by LC-MS/MS. 

Our method, remarkably, was able to detect and quantify HS (D0A0 and D0S0) in as few as 40,000 neurons from the TfR^mu/hu^ KI (herein referred to as WT) mice, although D0a4 was below LLOQ in WT mice (Figure S5). FACS-isolated neurons from WT mouse showed the lowest GAG levels relative to the other cell types, which could be partly due to technical difficulties in isolating intact neurons with long axons from adult mouse brains. This, however, should not affect the fold difference calculation between WT and *Ids* KO neurons as the isolation process was identical for both genotypes. Our ability to detect GAGs from a small number of neurons despite potential loss of neuronal GAGs during sample processing illustrates the superior sensitivity our new method to reliably measure GAGs in different types of FACS-isolated cells. From Table 3, we can see GAG accumulation in *Ids* KO animals in microglia was much higher (100-fold over WT) relative to astrocytes and neurons (5-15-fold over WT). This finding is consistent with a significant involvement of microglial activation in MPS II model mice [11]. In addition to FACS-isolated cells, this LCMS/MS method was also used to quantify GAGs in cell lines such as HEK293 and fibroblasts derived from MPS II patients (see Reference [11]).

#### 2.3.2. GAG Accumulation in *Ids* KO Mouse Model and Neuronopathic MPS II Patients

We sought to understand whether the proportions of the disaccharide species in brain tissue from MPS II model mice correlated with CSF GAGs, which would support the use of CSF GAG quantification as a biomarker for changes in patient brain GAGs. To study this, we performed GAGs analysis in the CSF, brain and liver from wildtype and *Ids* KO mice. We then extended these findings to patients with a study of human CSF from neuronopathic MPS II (nMPS II) patients and the age-matched controls.

The quantitation results in Table 3 show that CSF GAG disaccharides were in the 10 to 110 ng/mL range for individual GAG species in WT mice as well as non-MPS human controls. The sum of CSF GAGs were < 200 ng/mL, which is in agreement with published data for both WT mouse and non-MPS controls (Table 3). The concentrations of GAGs in both MPS II mouse model and nMPS II patients vs. controls are both around 10- to 20-fold higher than their healthy counter parts. In agreement with previously published work in MPS I mice [41], these results further highlight that there are differences in the relative abundance of various GAG species depending on the tissue type (brain, CSF or liver here). Specifically, the buildup of GAGs in mouse liver appears to be driven equally by HS and DS derived disaccharides. In contrast, in the CNS compartment (CSF, brain and FACS-isolated cells), GAG accumulation is primarily driven by the HS disaccharide, D0A0 (Table 3). Recent studies by Ullman et al. [11] and Bhalla et al. [42] furthermore demonstrated that CSF GAG levels in *Ids* KO mice were highly positively correlated with brain GAGs, and that our BBB-penetrating ERT (ETV:IDS) treatment significantly lowered both CSF and brain GAGs in the same dose-dependent fashion, suggesting that changes in CSF GAGs could well reflect their changes in the brain. This supports a promising application of CSF GAGs as disease relevant biomarker for future therapy development. 

For our analysis of HS and DS in human CSF, neuronopathic MPS II (nMPS II) patient samples were obtained from the Program for the Study of Neurodevelopment in Rare Disorders, University of Pittsburgh Medical Center. CSF GAGs in these patients were compared to that of age-matched controls and the measured concentrations can be found in Table 3. These data demonstrate that, even though these patients are on standard of care ERT (Elaprase™), there is a significant elevation of HS and DS in CSF relative to controls (Bhalla et al. 2020 [42]). These results support the finding that standard of care ERT does not address the CNS manifestation of MPS II [43].

## 3. Materials and Methods 

### 3.1. Animal and Preclinical Samples

#### 3.1.1. Animal Care 

All procedures in animals were performed in adherence to ethical regulations and protocols approved by Denali Therapeutic Institutional Animal Care and Use Committee. Mice were housed under a 12-hour light/dark cycle and had access to water and standard rodent diet (LabDiet® #25502, Irradiated) ad libitum. 

#### 3.1.2. Mouse Strains 

A previously described *Ids* KO mouse model on a B6 background was obtained from The Jackson Laboratories (JAX strain 024744)^2^. The TfR^mu/hu^ KI mouse line harboring the human TfR apical domain knocked into the mouse receptor was developed by generating a knock-in (into C57Bl6 mice) of the human apical TfR mouse line via pronuclear microinjection into single cell embryos, followed by embryo transfer to pseudo pregnant females using CRISPR/Cas9 technology. The donor DNA comprised the human TfR apical domain coding sequence, codon optimized for expression in mouse. The resulting chimeric TfR was expressed in vivo under the control of the endogenous promoter. A founder male from the progeny of the female that received the embryos was bred to wild-type females to generate F1 heterozygous mice. Homozygous mice were subsequently generated from breeding of F1 generation heterozygous mice [10] TfR^mu/hu^ male mice were bred to female *Ids* heterozygous mice to generate *Ids* KO; TfR^mu/hu^ mice [11]. All mice used in this study were males. For the study characterizing CSF, Brain and liver GAGs, mice were approximately 8.5 months of age at time of sample collection, whereas mice for the FACS study were approximately 4.5 months of age.

#### 3.1.3. Tissue and CSF Collection

For terminal sample collection, animals were deeply anesthetized via intraperitoneal (i.p.) injection of 2.5% Avertin. Animals were transcardially perfused with ice-cold PBS using a peristaltic pump (Gilson Inc. Minipuls Evolution) and the liver and brain were dissected and flash-frozen on dry ice. For CSF collection, a sagittal incision was made at the back of the animal’s skull, subcutaneous tissue and muscle was separated to expose the cisterna magna and a pre-pulled glass capillary tube was used to puncture the cisterna magna to collect CSF. CSF was transferred to a Low Protein LoBind Eppendorf tube and centrifuged at 12,700 rpm for 10 minutes at 4 °C. CSF was transferred to a fresh tube and snap frozen on dry ice. Lack of blood contamination in mouse CSF was confirmed by measuring the absorbance of the samples at 420 nm. 

CSF samples of healthy human subjects and pediatric MPS II patients with the demographics data were provided by Dr. Maria L. Escolar at University of Pittsburgh Medical Center. The human subject samples were approved (January 11th, 2012) by the Institutional Review Board of the University of Pittsburgh (PRO11050036).

### 3.2. Chemicals and Consumables

Acetonitrile (ACN), Optima LCMS Grade, 4 L (Catalog # A955-4, Thermo Fisher Scientific, Asheville, NC U.S.A.). Formic Acid (FA), Optima LCMS Grade (Catalog # A117, Thermo Fisher Scientific). Ammonium Formate, MS Grade (Catalog # 55674, Sigma, St Louis, MO, USA). Ammonium Formate, MS Grade (Catalog # 55674, Sigma, St Louis, MO, USA); Water, Optima LCMS grade 4L (Catalog # W6-4, Thermo Fisher Scientific) or 18.2 MΩ Reverse Osmosis (RO) Water. Bicinchoninic acid (BCA) assay (Pierce, Rockford, IL, USA). D0A0 ∆UA-GlcNAc(IV-A) (HD006); D0S0, ∆UA–GlcNS (IV-S) (HD005); D0a4 ∆UA–GalNAc,4S (∆UA–4S) (CD002); 4UA-2S-GlcNCOEt-6S (HD009) ultrapure (95%+) were purchased from Iduron (https://iduron.co.uk/) on custom order. Heparinases I, II, III and chondroitinase B. 30 kDa NWCO filter plate (Millipore, MSUN03010). ACQUITY UPLC BEH Amide 1.7 mm, 2.1×150 mm (Catalog # 186004802, Waters Corp., Milford, MA USA). Chondroitinase B 1.0 IU; 200mIU/ µL, Heparinase I 0.1 IU, 10mIU/ µL, Heparinase II 0.1 IU; 10mIU/ µL, Heparinase III 0.1 IU; 10mIU/µL (Galen/Iduron, UK https://iduron.co.uk).

### 3.3. Tissue or Fluid Processing for GAG Analysis 

50 mg tissue was homogenized in 750 µL water using the Qiagen TissueLyzer II for 3 min at 30Hz (×2). Homogenate was transferred to a 96-well deep plate and sonicated using a 96-tip sonicator (Q Sonica) for 10 × 1 second pulses (×2). Sonicated homogenates were spun at 2500× *g* for 30 minutes at 4 °C. The resulting lysate was transferred to a clean 96-well deep plate, and a BCA assay was performed to quantify total protein amounts. 10 ug of total protein lysate was used for liver and brain for subsequent HS/DS digestion. FACS-sorted cell pellets were resuspended in digest buffer before digestion. Digestion was carried out in a PCR plate in a total volume of 62 µL (all tissue lysates and biofluids). Lysates or body fluids (3 µL of mouse CSF, 5 µL of serum and 10 µL of urine) were mixed with Heparinases I, II, III (1.25 mIU each/sample; Iduron, UK) and Chondriotinase B (12.5 mIU/sample; Iduron, UK) in digestion buffer (111 mM NH4OAc, 0.11 mM CaOAc, pH 7.0) with internal standard mix of HS and DS (20 ng total per sample) for 3 h with shaking at 30 °C. After the digest, EDTA (final concentration of 2.5 mM) was added to each sample and the mixture was boiled at 95 °C for 10 min. The digested samples were spun at 3364× *g* for 5 minutes and samples were transferred to a cellulose acetate filter plate (Millipore, MSUN03010) and spun at 3364× *g* for 5 min. The resulting flow through was mixed with equal parts of acetonitrile in glass vials and analyzed by mass spectrometry as described above. 

### 3.4. FACS-Based CNS Cell Type Isolation 

To prepare a single cell suspension for sorting CNS cells, mice were perfused with PBS, brains dissected and processed into a single cell suspension according to the manufacturers’ protocol using the adult brain dissociation kit (Miltenyi Biotec 130-107-677). Cells were Fc blocked (Biolegend #101320, 1:100) and stained for flow cytometric analysis with Fixable Viability Stain BV510 (BD Biosciences #564406, 1:100) to exclude dead cells, CD11b-BV421 (BD Biosciences 562605, 1:100), CD31-PerCP Cy5.5 (BD Biosciences #562861, 1:100), O1-488 (Thermo/eBio #14-6506-82, 1:37.5), Thy1-PE (R&D #FAB7335P, 1:100), and EAAT2-633 (Alomone #AGC-022-FR, 1:50). Cells were washed with PBS/1% BSA and strained through a 100 μm filter before sorting CD11b+ microglia, EAAT2+ astrocytes, and Thy1+ neurons on a FACS Aria III (BD Biosciences) with a 100 μm nozzle. To achieve pure populations of astrocytes, microglia, and neurons negative gates were set to remove O1+ and CD31+ cells which are predominantly oligodendrocytes and endothelial cells respectively. Sorted cells were either pelleted or collected directly into lysis buffers, and then processed for downstream analysis including qRT-PCR, RNAseq, or glycomics as described in the relevant methods. 

### 3.5. Mass Spectrometry Analysis of GAGs 

Quantification of GAG levels in cells, fluids, and tissues was performed by liquid chromatography (Shimadzu Nexera X2 system, Shimadzu Scientific Instrument, Columbia, MD, USA) coupled to electrospray mass spectrometry (Sciex 6500+ QTRAP, Sciex, Framingham, MA, USA). For each analysis, sample was injected on a ACQUITY UPLC BEH Amide 1.7 mm, 2.1 × 150 mm column (Waters) using a flow rate of 0.6 mL/min with a column temperature of 55 °C. Mobile phases A and B consisted of water with 10 mM ammonium formate and 0.1% formic acid, and acetonitrile with 0.1% formic acid, respectively. An isocratic elution was performed with 80%B throughout the 8-min run. Electrospray ionization was performed in the negative-ion mode applying the following settings: curtain gas at 20; collision gas was set at medium; ion spray voltage at −4500; temperature at 450 °C; ion source Gas 1 at 50; ion source Gas 2 at 60. Data acquisition was performed using Analyst 1.6.3 or higher (Sciex) in multiple reaction monitoring mode (MRM), with dwell time 100(msec) for each species. Collision energy at −30; declustering potential at -80; entrance potential at −10; collision cell exit potential at -10. GAGs were detected as [M-H]^-^ using the following MRM transitions: D0A0 at m/z 378.1 > 87.0; D0S0 at m/z 416.1 > 138.0; D0a4 at m/z 458.1 > 300.0; D4UA-2S-GlcNCOEt-6S (HD009, Iduron Ltd., Manchester, UK) at m/z 472.0 (in source fragment ion) > 97.0 was used as internal standard (20ng/sample). Individual disaccharide species were identified based on their retention times and MRM transitions using commercially available reference standards (Iduron Ltd). GAGs were quantified by the peak area ratio of D0A0, D0S0, and D0a4 to the internal standard using Analyst 1.7.1 or MultiQuant 3.0.2 (Sciex). Reported GAG amounts were normalized to total protein levels as measured by a BCA assay (Pierce), and interpolated against a calibration curve.

### 3.6. Heparan Sulfate (HS) and Dermatan Sulfate (DS) Calibration Curves and QCs 

Pure standards for D0a4 (DS/CS), D0A0 (HS), and D0S0 (HS) were dissolved in acetonitrile: water 50/50 (*v*/*v*) to generate a 1 mg/mL stock. An eight-point dilution curve in matrix was generated, at 5, 10, 20, 100, 1000, 5000, 9000 to 10000 ng/mL. Three levels of QC samples were prepared at 15, 300 and 7500 ng/mL. The calibration curve standards and QCs went directly to enzymatic digestion, together with biological samples, but without adding enzyme, and the following steps and run by LC-MS/MS as described in Section 3.5. Additionally, one lot of pooled CSF was used as Matrix QC and digested at the same time with study samples to monitor the digestion consistency.

## 4. Conclusions

We developed a robust and highly sensitive method to quantify sulfated glycosaminoglycans (GAGs) in various matrices. This method enabled accurate quantification of HS and DS GAGs in pre-clinical samples and provided the sensitivity to quantify GAGs in as low as 3 µL CSF from mice, as well as in low volume (20 µL) of CSF from nMPS II patients and non-MPS pediatric controls. In addition, the high sensitivity of this method allowed for quantifying GAGs in individual cell populations sorted from mouse brain tissue. Using this method, we detected significant accumulation of GAGs in an MPS II mouse model and nMPS II patients where the CSF and brain GAGs accumulation were positively correlated and driven by same species, supporting the feasibility of using GAGs in CSF as proxy biomarker for brain GAGs. In addition, the ability to measure individual GAGs species presents a broad and generic application of this method to different MPS disorders where more specific GAGs need to be measured, for example, in MPS III patients only HS is elevated [18] whereas in MPS VI only DS is elevated [44].

## Figures and Tables

**Table 1:** Fit-for-purpose qualification results for Heparan and Dermatan Sulfate Disaccharides in CSF for calibration curves in Figure 3.

Parameter	Qualification Result	
Range of Standard Curve	D0A0: 5 to 10,000 ng/mL; D0S0: 10 to 10,000 ng/mL; D0a4: 5 to 10,000 ng/mL	
LLOQ	D0A0: 10 ng/mL; D0S0: 10 ng/mL; D0a4: 5 ng/mL	
Inter-assay precision (% CV) and accuracy (% bias) for standards	% CV: D0A0: 5.3% to 9.6% D0S0: 5.0% to 10.1% D0a4: 6.2% to 16.9%	% Bias: D0A0: −4.4% to 3.5% D0S0: −2.7% to 3.7% D0a4: −4.7% to 3.1%
Intra-assay precision (%CV) and accuracy (% bias) for QCs (*n* = 3)	% CV: D0A0: 1.6% to 9.4% D0S0: 3.0% to 9.2% D0a4: 2.6% to 6.3%	% Bias: D0A0: −7.4% to 9.7% D0S0: −11.9% to 6.0% D0a4: −10.9% to 15.0%
Inter-assay precision (% CV) and accuracy (% bias) for QCs (*n* = 3)	% CV: D0A0: 5.5% to 8.7% D0S0: 5.9% to 10.1% D0a4: 5.4% to 10.5%	% Bias: D0A0: −2.6% to 3.3% D0S0: −6.0% to −0.1% D0a4: −4.7% to 8.0%
Inter-assay precision (% CV) for Pooled CSF Matrix QC (*n* = 3)	% CV: D0A0: 7.9% D0S0: 7.4% D0a4: 10.5%	
QC benchtop stability	Benchtop stability was confirmed for 24 h at room temperature	
Injection carryover	No carryover observed	

Abbreviations: LLOQ = lower limit of quantitation; QC = quality control; CV = coefficient of variation.

**Table 2:** A summary of the CSF GAG measurement in MPS patients and animal models.

Year	GAG Species	Approach	Assay Principle	LOD; LOQ/LLOQ (ng/mL)	Patient/Disease Model	Concentration (ng/mL)	CSF Volume	Ref
1970	Hexuronic acid (acidic GAGs)	Acid hydrolysis	Carbazole reaction Colorimetry	n/a	Hunter-Hurler’s syndrome	750–4000 (**MPS**) **^a^**; 100 **(HC) ^b^**	12–15 mL	[12]
1981	Uronic acid: disaccharides	Acid hydrolysis	Carbazole reaction Colorimetry	n/a	MPS IIIB (Sanfilippo B syndrome)	850 (**MPS**); 200 **(HC)**	<10 mL	[37]
2008	Sulfated GAGs (intact)	Dye binds to sulfated GAGs	Dimethyl methylene blue assay	n/a	MPS IH (Hurler syndrome)	13,300 (**MPS**); 10,300 **(HC)**	mLs	[38]
2011	HS, DS: disaccharides	Acid hydrolysis	LC-MS/MS	200 (LOQ)	MPS IH (Hurler syndrome)	HS 7000–11000; DS 1100–2600 (**MPS**); HS < 380; DS < 240 **(HC)**	25 µL	[17]
2015	DS (intact)	DS stimulates heparin cofactor II-mediated thrombin inhibition	ELISA thrombin activity assay with chromogenic substrate S-2238^TM^	37 (LOD intact GAG)	MPS II (Hunter syndrome)	800–2360 w CI **^c^** (**MPS**), 380–1180 w/o CI ^d^ (**MPS**); <200 **(HC)**	100 µL	[39,40]
2016	D2S6, D0S6, D2S0, D0S0, D0A0, D0A6, disaccharides	13C-labeled 4 butylaniline labeling	LC-MS/MS	50 (LOQ)	MPS IIIA patient	2500-3500 (**MPS**); 200 **(HC)**	50 µL	[18]
2018	HS, DS: disaccharides	Acid hydrolysis	LC-MS/MS	25 (LOD)	MPS II disease model mice	1500–6000(***Ids* KO)** ^e^; n/a **(WT)**^f^	2 µL	[13]
2018	DS (DS4S/DS6S) disaccharides	Chondrotinase B (enzyme)	LC-MS/MS	20 (LLOQ) for DS polysaccharides	MPS II patients	~900 (**MPS**); 80 **(HC)**	100 µL	[14]
2019	HS, DS: disaccharides	Acid hydrolysis	LC-MS/MS	25 (LLOQ)	MPS II patient	DS 800–1900; HS 4200–7900 w CI; 2600–4600 w/o CI (**MPS**); 360 (**HC**)	n/a	[15]
2019	D0S6, D2S0, D0S0 D0A6, disaccharides	Enzymatic digestion, Aniline labeling	LC-MS/MS	200 (LOQ)	MPS IIIA patient	n/a (**MPS**); 250 **(HC)**	25 µL	[20]
2020	D0A0, D0S0, D0a4 HS and DS disaccharides	Enzymatic digestion	LC-MS/MS	3(LOD); 5-10 (LLOQ)	MPS II disease model mice MPS II patient	1800–6000 (***Ids* KO**); <200 **(WT)** ^f^ ~1000 (**MPS**); <100**(HC)**	3 µl (mouse) 20 µL (human)	This paper

GAGs concentration of **^a^**: Various MPS patients (**MPS**), **^b^**: Human Healthy Control (**HC**), **^c^**: w CI: with cognitive impairment, **^d^**: w/o CI, without CI **^e^**: IDS knockout (***Ids* KO**) mice **^f^**: TfR^mu/hu^ KI (**WT**) mice. LOD: Limit of Detection. LOQ: Limit of Quantification.

**Table 3:** HS and DS concentration in FACS-isolated neurons and microglia, CSF, brain, and liver in mice, and CSF of healthy human subjects and MPS II patients (Mean ± SEM).

Sample Type	D0A0 (HS)	D0S0 (HS)	D0a4 (DS)	Sum GAGs
FACS neuron (WT; *n* = 4) ^a^	0.4 ± 0.07	4 ± 0.8	0.1 ± 0.02	5 ± 0.9
FACS neuron (*dsS* KO; *n* = 6) ^a^	4 ± 0.8 (***10x***) ^b^	20 ± 5 (***5x***)	1 ± 0.4 (***10x***)	25 ± 5 (***5x***)
FACS microglia (WT; *n* = 4) ^a^	6 ± 0.8	40 ± 10	0.8 ± 0.1	47 ± 11
FACS microglia (*Ids* KO; *n* = 6) ^a^	2e3 ± 400 (***333x***)	3e3 ± 400 (***75x***)	210 ± 40 (***262x***)	5e3 ± 800 (***106x***)
FACS astrocyte (WT; *n* = 4) ^a^	0.4 ± 0.08	5 ± 2	0.09 ± 0.01	5 ± 2
FACS astrocyte (*Ids* KO; *n* = 6) ^a^	10 ± 2 (***25x***)	70 ± 20 (***14x***)	4 ± 1 (***44x***)	81± 22 (***14x***)
CSF (WT; *n* = 7) ^c,e^	109 ± 2	28 ± 2	6 ± 0.3	143 ± 2
CSF (*Ids* KO; *n* = 7) ^c,e^	1.2e3 ± 96 (***11x***)	217 ± 8 (***8x***)	131 ± 19 (***21x***)	1.5e3 ± 45 (***11x***)
Brain (WT; *n* = 7) ^d^	331 ± 14	117 ± 8	85 ± 8	533 ± 28
Brain (*Ids* KO; *n* = 7) ^d^	8.2e3 ± 307 (***22x***)	1.0e3 ± 39 (***5x***)	734 ± 65 (***8x***)	1.0e4 ± 364 (***13x***)
Liver (WT; *n* = 7) ^d^	203 ± 13	75 ± 6	175 ± 24	452 ± 40
Liver (*Ids* KO; *n* = 7) ^d^	2.8e4 ± 3e3 (***139x***)	5.0e3 ± 580 (***67x***)	9.0e3 ± 1.2e3 (***51x***)	4.2e4 ± 4.8e3 (***93x***)
Healthy Human CSF (*n* = 25) ^e^	35 ± 3	13 ± 1	10 ± 0.8	59 ± 4
MPS II patient CSF (*n* = 6) ^e,f^	301 ± 39 (***9x***)	98 ± 16 (***8x***)	522 ± 117 (***52x***)	921 ± 147 (***16x***)

^a^: HS and DS disaccharide levels were normalized to cell number and depicted as pg/k cells. Please note that the HS and DS quantification in FACS-isolated cells was based on peak area to I.S. area (relative quantification) and not by a calibration curve. ^b^: x refers to fold changes compared to WT or healthy. ^c^: HS and DS disaccharide levels in CSF were normalized to volume and depicted as ng/mL; ^d^: HS and DS value in tissue is normalized to total protein and depicted as ng/mg of tissue lysate; ^e^: CSF HS and DS levels were quantified based on the fit-for-purpose qualified preclinical assay as we described in Section 2.2; ^f^: MPS II patients are on ERT at the time of sample collection.

## Supplementary Materials

Supplementary materials can be found at https://www.mdpi.com/1422-0067/21/15/5449/s1.

## Abbreviations

BBB	Blood–Brain Barrier
IDS	Iduronate 2-Sulfatase
GAG	Glycosaminoglycan
LC-MS/MS	Liquid Chromatography-Tandem Mass Spectrometry
HS	Heparan Sulfate
DS	Dermatan Sulfate
CSF	Cerebrospinal Fluid
FACS	Fluorescence-Activated Cell Sorting
TfR	Transferrin Receptor
LOD	Limit of Detection
LLOQ	Low Limit of Quantification
MPS II	Mucopolysaccharidosis II
ETV	Enzyme Transport Vehicle
D0A0	4,5 unsaturated uronic acid residue-N-acetyl glucosamine, HS ∆UA-GluNAc
D0S0	4,5 unsaturated uronic acid residue-glucosamine-N-sulfate, ∆UA-GluNS
D0a4	4,5 unsaturated uronic acid residue-N-acetyl galactosamine, 4-O-sulfate, ∆UA-GalNAc4S
DQC	Dilution Quality Control
HQC	High Quality Control
LQC	Low Quality Control
MQC	Medium Quality Control
