# Supplementary material for: High-Throughput Liquid Chromatography–Tandem Mass Spectrometry Quantification of Glycosaminoglycans as Biomarkers of Mucopolysaccharidosis II
Source: Int J Mol Sci. 2020 Jul 30;21(15):5449. doi: 10.3390/ijms21155449 (PMC7432392; doi:10.3390/ijms21155449)
Supplement: Supplementary file 1 [file ijms-21-05449-s001.pdf]

International Journal of
Molecular Sciences

MDPI

Article

High-throughput Liquid Chromatography-Tandem Mass Spectrometry Quantification of Glycosaminoglycans as Biomarkers of Mucopolysaccharidosis II

Junhua Wang†,*, Akhil Bhalla†, Julie C. Ullman, Meng Fang, Ritesh Ravi, Annie Arguello, Elliot Thomsen, Buyankhishig Tsogtbaatar, Jing L. Guo, Lukas L. Skuja, Jason C. Dugas, Sonnet S. Davis, Suresh B. Poda§, Kannan Gunasekaran, Simona Costanzo, Zachary K. Sweeney, Anastasia G. Henry, Jeffrey M. Harris, Kirk R. Henne and Giuseppe Astarita

Denali Therapeutics, Inc., 161 Oyster Point Blvd., South San Francisco, CA 94080. jwang@dnli.com (J.W.); bhalla@dnli.com (A.B.); ullman@dnli.com (J.C.U.); fang@dnli.com (M.F.); ravi@dnli.com (R.R.); arguello@dnli.com (A.A.); thomsen@dnli.com (E.T.); tsogtbaatar@dnli.com (B.T); guo@dnli.com (J.L.G); skuja@dnli.com (L.L.S.); dugas@dnli.com (J.D.); davis@dnli.com (S.D.); suresh_poda@eisai.com (S.P.); kannan@dnli.com (K.G.); costanzo@dnli.com (S.C.); zachary.sweeney@yahoo.com (Z.S.); henry@dnli.com(A.H.); jharris@dnli.com(J.H.); henne@dnli.com (K.H.); gastarita@gmail.com (G.A.)

† Those authors contributed equally.

§ Present Address: Eisai G2D2, 35 Cambridge park Drive, Cambridge, MA 02141

* Correspondence: jwang@dnli.com (J.W.)

Received: date; Accepted: date; Published: date

Appendix A – Supplemental data

Table S1. Acquisition parameters and retention time (RT) for GAG species on QTRAP 6500+.

Figure S1. HS and DS disaccharides by acid hydrolysis.

Figure S2. Methanolysis and Butanolysis LC-MS/MS of HS and DS in standard and CSF.

Figure S3. LC-MS and high-resolution MS/MS of HS by butanolysis using both butanol (B) and deuterated butanol (B-d10).

Figure S4. LC-MS and high-resolution MS/MS of DS by methanolysis using methanol-d4.

Figure S5. LC-MSMS of DS (D0a4), HS (D0A0 and D0S0) in 40,000 FACS isolated neurons.

Figure S6. HS and DS released by Enzymatic digestion using Heparitinase or chondroitinase B.

Table S1. Acquisition parameters and retention time (RT) for GAG species on QTRAP 6500+.

GAG Name	Internal Standard	RT (min)	Q1 m/z	Q3 m/z	Time (msec)	CE	DP
D0A0	4UA-2S-GlcNCOEt-6S	4.02	378.1	87.0	50	-30	-80
D0S0	4UA-2S-GlcNCOEt-6S	4.70	416.1	138.0	50	-30	-80
D0a4	4UA-2S-GlcNCOEt-6S	4.16	458.1	300.0	50	-30	-80
4UA-2S-GlcNCOEt-6S	N/A	4.3	472.0	97.0	50	-28	-80

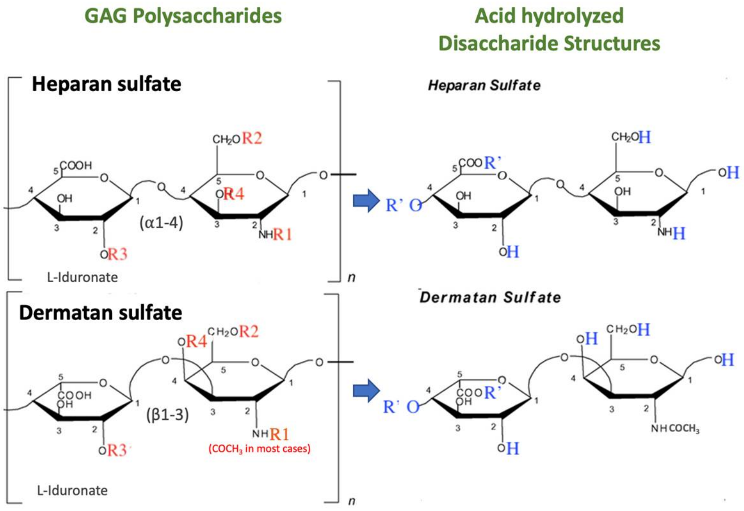

Disaccharides formula and masses

Alcohol	R'	Formula	[M+H]+1	MRM
**Heparan sulfate**				
methanol	CH3-	C14H25NO11	384.1500	384/162
ethanol	C2H5-	C16H29NO11	412.1813	412/162
butanol	C4H9-	C20H37NO11	468.2439	468/162
**Dermatan sulfate**				
methanol	CH3-	C16H27NO12	426.1606	426/236
ethanol	C2H5-	C18H31NO12	454.1919	454/236
butanol	C4H9-	C22H39NO12	510.2545	510/236

Figure S1. HS and DS disaccharides by acid hydrolysis. The hydrolysis with either methanol, ethanol or butanol depolymerization could reduce the chemical diversity of GAG species, for example, HS or CS/DS to a corresponding single small molecule disaccharide through the desulfation/deacetylation and dialkylation reactions. The structure, formula, detection mass in positive mode and the MRM are listed. R1-R4 in red are one of the following H, AC or SO^{3-} groups; R' in blue corresponds to the alcohol group that is used with acid for the hydrolysis reaction, and could be Me, Et, or Bu. After hydrolysis, the R is eliminated and R' is generated depending on the alcohol, the diversity is reduced to uniform product in this way.

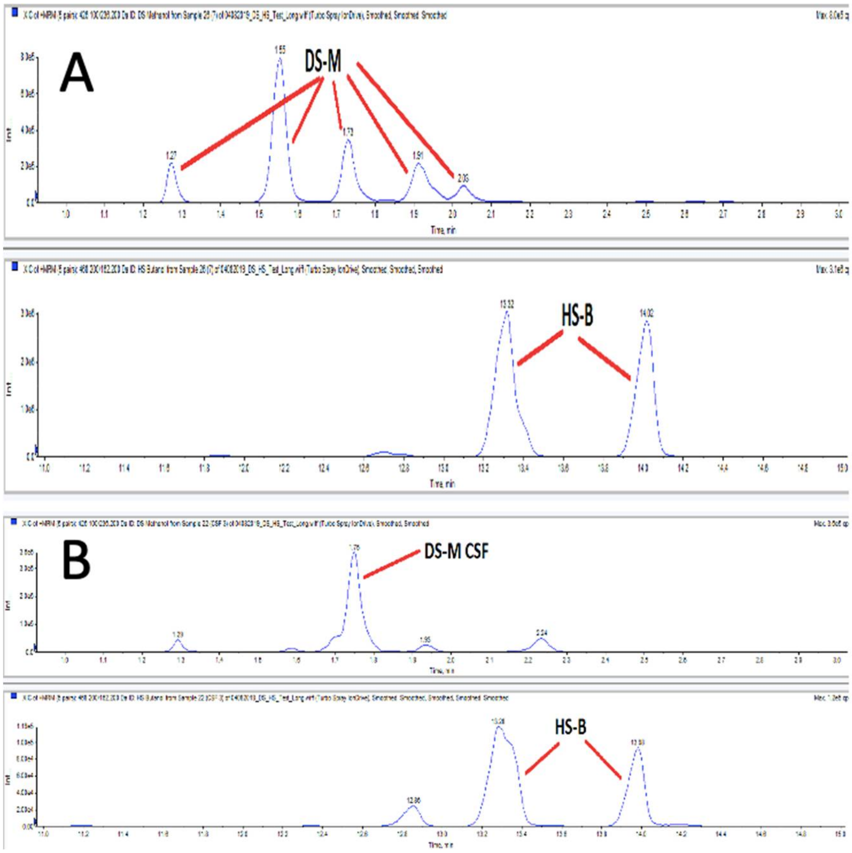

Figure S2. Methanolysis and Butanolysis LC-MS/MS of HS and DS in standard and CSF. (A). Standard. (B) CSF. Methanolysis Method: Sample preparation: 10 µL of fluid was evaporated to dryness. 200 µL methanolic HCL and 10 µL 2,2-dimethoxypropane was added to each well. Incubated at 65 °C for 75 min, evaporated to dryness and reconstituted with water 0.1% formic acid. Butanolysis Method: Sample preparation: 10 µL of fluid was evaporated to dryness. 100 µL of 3 N HCL in N-Butanol was added to each tube. Incubated at 65 °C for 25 min (HS) and 90 °C for 60 min (DS), evaporated to dryness and reconstituted with water/acetonitrile 1/1. LC-MS/MS Method: Mobil Phase A: Water 20 mM Ammonium formate, Mobil Phase B: Acetonitrile 0.1% formic acid. Column-Supelco Ascentis RP-Amide (10cm x 4.6 mm, 3 µm). Gradient- 5% B hold 30 seconds, ramp to 95% over 4 minutes. Mass Spec: Sciex QTRAP 5500. MRM: for HS-B: *m*/*z* 468 > 162 (positive ion), for DS-M: m/z 426 > 236 (positive ion).

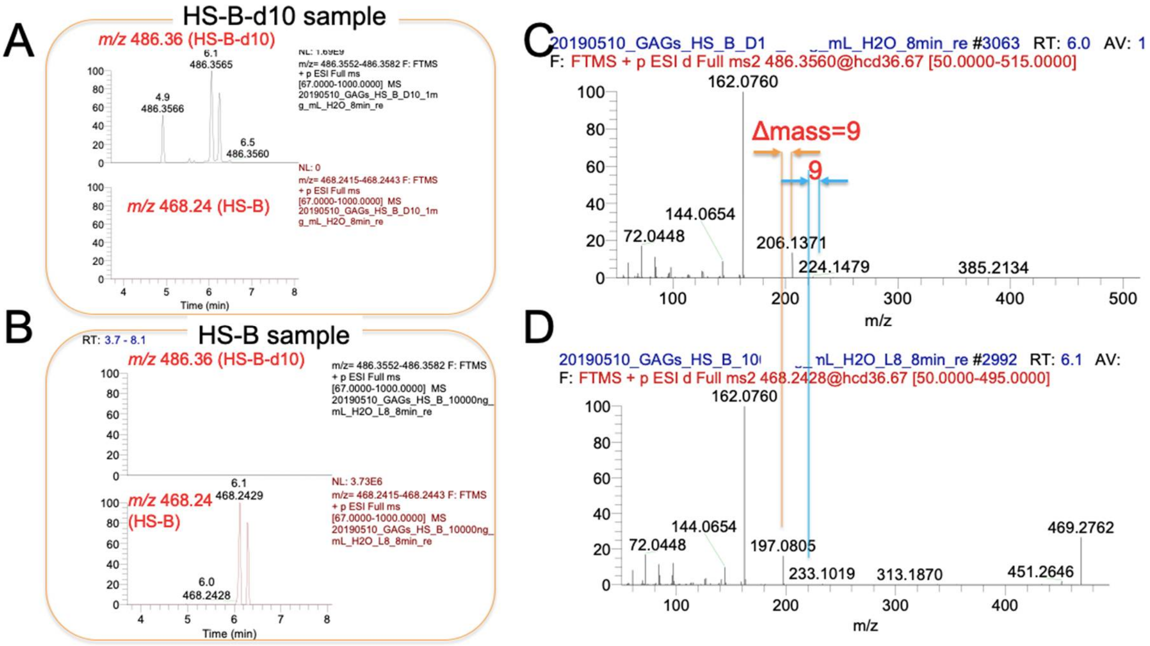

Figure S3. LC-MS and high resolution MS/MS of HS by butanolysis using both butanol (B) and deuterated butanol (B-d10). A) HS-B-d10 and B) HS-B peaks. Two peaks were detected for each sample. C) MS/MS for HS-B-d10, and D) MS/MS of HS-B. Both spectra confirmed the identities of the reactants. The HS-B spectrum verified the parent/dominant fragment masses are 468/162, which matches to the listing in Figure S3. LC-MS/MS Method: Mobil Phase A: Water 20 mM Ammonium formate, Mobil Phase B: Acetonitrile 0.1% formic acid. Column-Supelco Ascentis RP-Amide (10cm x 4.6 mm, 3 μm). Gradient- 5% B hold 30 seconds, ramp to 95% over 4 minutes.
Mass Spec: Thermo Scientific. Q Exactive operated under positive ion mode.

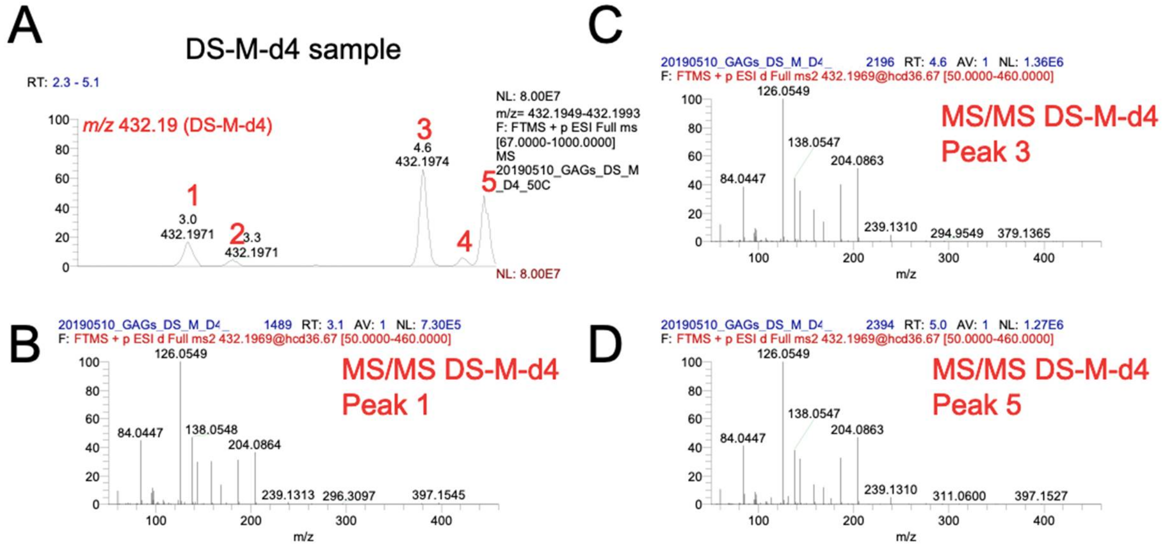

Figure S4. LC-MS and high resolution MS/MS of DS by methanolysis using methanol-d4. A) Five peaks observed for the DS-M-d4. B-D) MS/MS for the DS-B-d4 peak 1, 3, 5. The spectra are identical and they confirmed the identities of the reactants. LC-MS/MS Method: Mobil Phase A: Water 20 mM Ammonium formate, Mobil Phase B: Acetonitrile 0.1% formic acid. Column-Supelco Ascentis RP-Amide (10cm x 4.6 mm, 3 μm). Gradient- 5% B hold 30 seconds, ramp to 95% over 4 minutes.
Mass Spec: Thermo Scientific. Q Exactive operated under positive ion mode.

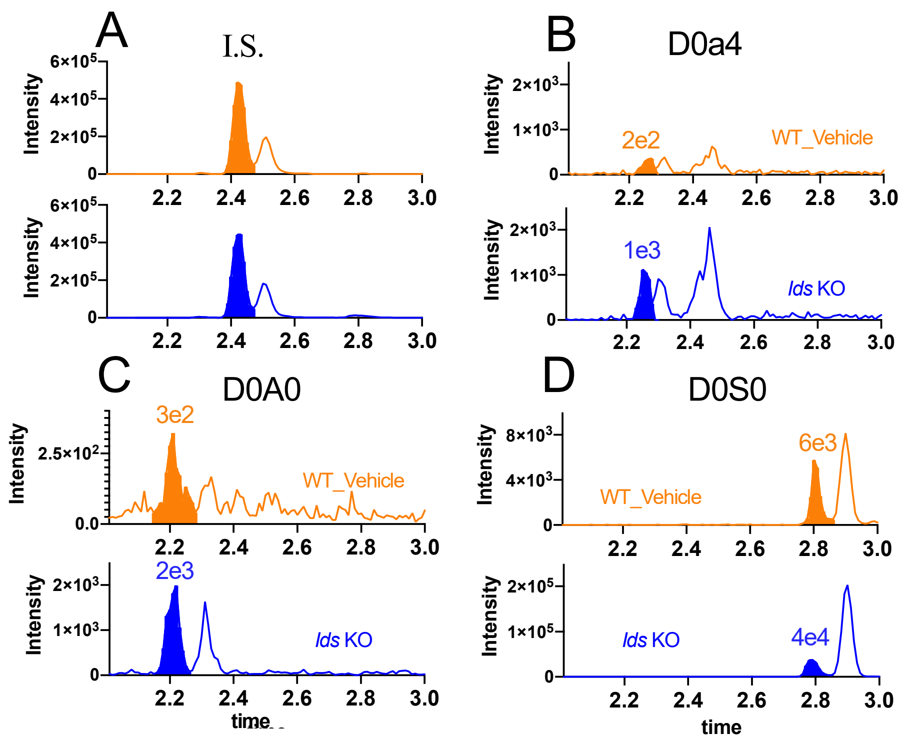

Figure S5. LC-MSMS of DS (D0a4), HS (D0A0 and D0S0) in 40,000 FACS isolated neurons. D0A0 and D0S0 were quantifiable in 40,000 neurons and D0a4 signal was below LLOQ. Orange trace = WT sample and blue trace = *Ids*:KO sample. LC was performed using a 4.5-minute gradient programmed as follows: 0.0–0.5 minutes at 80%B, 0.5–3.5 minutes from 80%B to 50%B, 3.5–4.0 minutes 50%B to 80%B, 4.0-4.5 minutes hold at 80%B. LC flow rate of 0.55 mL/min, column temp at 55°C. Column: ACQUITY UPLC BEH Amide 1.7mm, 2.1×150 mm (Catalog # 186004802, Waters Corp., Milford, MA USA). Mass Spec: Sciex QTRAP 6500+.

Enzymatically Cleaved Disaccharide structures

Heparan sulfate

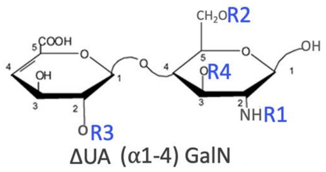

Dermatan sulfate

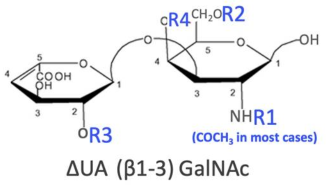

Disaccharides formula and masses

ΔDiHS/DS	R1	R2	R3	R4	DSC	Sigma	Structure	Formula	$[M\text{-}H]^{-1}$	MRM
**Major Heparan sulfate product species**										
-0S	Ac	H	H	H	D0A0	IV-A	ΔUA-GlcNAc	C14H21NO11	378.1046	378/87
-NS	SO^{3-}	H	H	H	D0S0	IV-S	ΔUA-GlcNS	C12H19NO13S	416.0509	416/138
-6S	Ac	SO^{3-}	H	H	D0A6	II-A	ΔUA-GlcNAc6S	C14H21NO14S	458.0615	458/97
-US	Ac	H	SO^{3-}	H	D2A0	III-A	ΔUA2S-GlcNAc	C14H21NO14S	458.0615	458/97
-(6,N)S	SO^{3-}	SO^{3-}	H	H	D0S6	II-S	ΔUA-GlcNS6S	C12H19NO16S2	496.0077	496/416
-(U,N)S	SO^{3-}	H	SO^{3-}	H	D2S0	III-S	ΔUA2S-GlcNS	C12H19NO16S2	496.0077	496/416
-(U,N,6)S	SO^{3-}	SO^{3-}	SO^{3-}	H	D2S6	I-S	ΔUA2S-GlcNS6S	C12H19NO19S3	575.9645	576/97
-(N,6)S	Ac	SO^{3-}	SO^{3-}	H	D2A6	I-A	ΔUA2S-GlcNAc6S	C14H21NO17S2	538.0183	538/97
**Major Dermatan sulfate product species**										
-0S	Ac	H	H	H	D0a0		ΔUA-GalNAc	C14H21NO11	378.1046	378/175
-4S	Ac	H	H	SO^{3-}	D0a4		ΔUA-GalNAc4S	C14H21NO14S	458.0615	458/300
-6S	Ac	SO^{3-}	H	H	D0a6		ΔUA-GalNAc6S	C14H21NO14S	458.0615	458/97
-US	Ac	H	SO^{3-}	H	D2a0		ΔUA2S-GalNAc	C14H21NO14S	458.0615	458/97
-(N,4)S	Ac	H	SO^{3-}	SO^{3-}	D2a4		ΔUA2S-GalNAc4S	C14H21NO17S2	538.0183	538/458
-(N,6)S	Ac	SO^{3-}	SO^{3-}	H	D2a6		ΔUA2S-GalNAc6S	C14H21NO17S2	538.0183	538/458
-(4,6)S	Ac	SO^{3-}	H	SO^{3-}	D0a10		ΔUA-GalNAc4S6S	C14H21NO17S2	538.0183	538/458

Figure S6. HS and DS released by Enzymatic digestion using Heparinase or Chondroitinase B. The disaccharide code (DSC) (according to Lawrence [1]), structure nomenclature, formula, accurate mass and ion transition (MRM) are listed for the major HS and DS species. Products maintain both HS and DS original chemical modifications and the structural diversities.

Reference:

[1] Lawrence, R.; Lu, H.; Rosenberg, R.D.; Esko, J.D.; Zhang, L. Disaccharide structure code for the easy representation of constituent oligosaccharides from glycosaminoglycans. *Nat. Methods* **2008**, 5, 291-292
